# Therapeutic prospects and potential mechanisms of Prdx6: as a novel target in musculoskeletal disorders

**DOI:** 10.3389/fphys.2025.1524100

**Published:** 2025-04-17

**Authors:** Hong Sun, Chao Xu, Zhilin Xiong, Miao Liu, Xu Ning, Yong Zhuang

**Affiliations:** ^1^ Department of Orthopaedics, The Affiliated Hospital of Guizhou Medical University, Guiyang, China; ^2^ School of Clinical Medicine, Guizhou Medical University, Guiyang, China

**Keywords:** peroxiredoxin 6, Prdx6, musculoskeletal disorders, MSDs, cell death, oxidative stress, inflammation

## Abstract

With the global population aging, musculoskeletal disorders (MSDs) have posed significant physical and psychological health challenges for patients as well as a substantial economic burden on society. The advancements in conservative and surgical interventions for MSDs have been remarkable in recent years; however, the current treatment modalities still fall short of meeting the optimal requirements of patients. Recently, peroxiredoxin 6 (Prdx6) has gained considerable attention from researchers due to its remarkable antioxidative, anti-inflammatory, and anti-apoptotic properties. It has been found that Prdx6 is involved in multiple system diseases, including MSDs; however, the exact role of Prdx6 in MSDs is still lacking. This study aimed to summarize the structure, regulatory mechanism, and potential function of Prdx6. These findings may demonstrate Prdx6 as a novel target for inhibiting the advancement of MSDs.

## 1 Introduction

The musculoskeletal system is a fundamental physiological structure that facilitates the movement, support, and defense mechanisms of the human body (Bersini et al., 2016). It encompasses articulations, musculature, osseous structures, cartilage, and tendons (Huang et al., 2020); impairment of these components contributes to the pathogenesis of musculoskeletal disorders (MSDs). MSDs are a group of prevalent diseases characterized by a significant decline in muscle strength and quality, bone volume and quality, cartilage thickness, and intervertebral disc (IVD) integrity with the progression of aging (Song et al., 2023). The common conditions among them include osteoporosis (OP), osteoarthritis (OA), and intervertebral disc degeneration (IVDD) (Song et al., 2023; Zhang et al., 2020). The primary manifestations of these diseases include pain, inflammatory arthritis, musculoskeletal injury sequelae, impaired physical function, and disability (Briggs et al., 2018). It has been discovered that the development of MSDs is closely associated with factors including aging, oxidative stress, processes related to inflammation, and apoptosis (Zhang X. et al., 2024; Li et al., 2021a; Jiang et al., 2022). The prevalence of MSDs is increasing as the global population ages, with MSDs currently ranking as the leading cause of disability worldwide, placing significant strain on global health and social security systems (Chen et al., 2022). Despite the completion of numerous research projects and significant advancements in recent years, a comprehensive understanding of the etiology of MSDs remains elusive, and there is still a dearth of effective clinical interventions aimed at impeding or halting their progression (Briggs et al., 2018; Wen et al., 2023; Han et al., 2022; Liang et al., 2024). So now, The pursuit of novel treatment targets for MSDs is imperative in order to alleviate the suffering of affected individuals and mitigate the financial burden on society as a whole.

The Peroxiredoxins (Prdxs) family represents a crucial group of cellular anti-oxidative stress molecules with significant physiological importance (Hall et al., 2011). Eukaryotic Prdxs, under normal physiological conditions, exhibit the ability to effectively reduce approximately 100% of cytoplasmic hydrogen peroxide (H_2_O_2_) and around 90% of mitochondrial H_2_O_2_, thereby playing a pivotal role in antioxidant defense mechanisms (Hall et al., 2011). In mammals, there are six known peroxiredoxin varieties, namely, normal 2-Cys (Prdx1-4), atypical 2-Cys (Prdx5), and 1-Cys (Prdx6), which are classified based on the presence of cysteine residues in the active core and their respective catalytic mechanisms (Hall et al., 2011; Sharapov et al., 2022; Xue et al., 2024). Prdx6, a significant member of the peroxiredoxin family, possesses phospholipase, lysophosphatidylcholine acyltransferase (LPCAT), and peroxidase activities (Fisher, 2018). As the 1-Cys member of the Prdx family, Prdx6 distinguishes itself from other family members by possessing only one conserved cysteine residue, and operating in its unique catalytic cycle, which operates independently without the involvement of thioredoxin as a reducing agent (Parsonage et al., 2005; Zhou S. et al., 2016). It exhibits distinct subcellular localization compared to other members of the family (Lee, 2020; Fisher, 2019). Moreover, owing to its multifunctional nature, Prdx6 exhibits potential for mitigating H_2_O_2_, alkyl hydroperoxides (ROOH), peroxynitrite (ONOO^−^), and phospholipid peroxides (PLOOH) (Sharapov et al., 2022; Xue et al., 2024; Fisher, 2011; Fisher, 2017). Prdx6 is distinctive among other Prdxs in the following Table 1.

**TABLE 1:** The characteristics of Prdxs members.

Members of the Prdxs family	Cysteine residues	The physiological reductant	Substrate binding	LPCAT and PLA2 activity	Subcellular localization
Prdx1-4	CR and CP are located in different subunits	Thioredoxin	H_2_O_2_, ROOH, ONOO-	None	Nucleus, cytosol, mitochondria reticulum (ER) and the cytosol
Prdx5	The CR is in the samesubunit as the CP	Thioredoxin	H_2_O_2,_ ROOH, ONOO-	None	Cytosol, peroxisomes, and mitochondria
Prdx6	Absence of CR	GSH, GST	H_2_O_2_, ROOH, ONOO−, PLOOH	Positive	Cytosol(primarily), lysosomes and lysosomal related organelles

LPCAT, LPC acyl transferase; PLA2, phospholipase A2 activity; -Cys, cysteine residue; CR, “resolving” cysteine; Cp, peroxidatic Cys, GSH, Glutathione ; GST, GSH S-transferase; ROOH, alkyl hydroperoxides; ONOO-, peroxynitrite; PLOOH, peroxides of phospholipids.

It has been demonstrated that Prdx6 plays a pivotal role in antioxidant defense, cellular metabolism regulation, inflammatory signaling modulation, and the maintenance of lipid peroxidation repair (Xue et al., 2024). According to research, Prdx6 might be suggested as a novel therapeutic target for the treatment and prevention of metabolic disorders linked to accelerated aging (Pacifici et al., 2020). Various systemic disorders are known to be influenced by Prdx6 in their onset and progression, including MSDs (Soriano-Arroquia et al., 2021; Bian et al., 2024; Xiong et al., 2023; Lysikova et al., 2024; Peng et al., 2024; Bumanlag et al., 2022; Chhunchha et al., 2023). However, the precise molecular mechanism underlying Prdx6 in MSDs remains poorly understood. This review comprehensively summarizes the molecular composition, expression regulation, and primary functions of Prdx6. And we postulate that Prdx6 may play a pivotal role in the pathogenesis and progression of MSDs through oxidative stress, inflammation, and aberrant cell death. In conclusion this review attempts to offer fresh perspectives on prdx6’s potential as a novel therapeutic target in MSDs.

## 2 Overview of Prdx6

### 2.1 Structure of Prdx6

The Prdxs family comprises small proteins ranging in size from 22 to 27 KDa; among these, native Prdx6 is a protein with a molecular weight of 26 KDa (Patel and Chatterjee, 2019). The human Prdx6 protein consists of 224 amino acids and can be categorized into two distinct domains: a larger N-terminal domain (residues 1–174) that exhibits a thioredoxin fold, comprising four-stranded β-sheets (β3, β4, β6, and β7), along with three lateral α-helices (α2, α4, and α5); and a smaller C-terminal domain (residues 175–224) composed of three β strands and one α-helix, the extended helix a5 and the underlying loop establish its connection to N-terminal domain (Choi et al., 1998). The active center of Prdx6 is primarily involved in several fundamental sequences: the peroxidase depend on Cys47 (Chen et al., 2000); the catalytic triad of H26, S32, and D140 dominates PLA2 activity (Manevich et al., 2007); and the LPCAT active domain consists of the 26HxxxxD31 sequence (Fisher et al., 2016; Jia et al., 2023). Consequently, the enzymatic activity of Prdx6 is relatively autonomous, leading to distinct impacts on enzyme function as a result of mutations at various sites (Jia et al., 2023). The oligomerization of Prdx6 undergoes changes in response to different oxidation states, which is closely linked to its functional role. The most striking difference that the crystal structures of human Prdx6 in reduced (SH) and sulfinic acid (SO_2_H) forms compared to the oxidized form is residues Leu114-Arg132, which is the loop connecting α4 and β6 (Kim et al., 2016).

**FIGURE 1:**
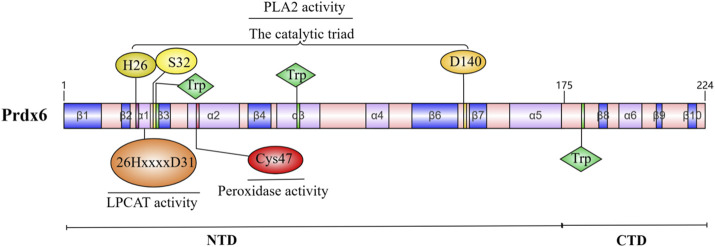
Molecular schematic of Prdx6 and its enzymatic major active site.

Prdx6 is encoded by an 11,542 basepair gene consisting of five exons located on human chromosome 1, which exhibits high conservation among human genes and demonstrates up to 95% identity when comparing the amino acid sequences of Prdx6 proteins in humans, mice, and rats (Xue et al., 2024; Fisher, 2011). These findings suggest that Prdx6 plays a pivotal role in mammalian cell metabolism. A schematic of the Prdx6 molecule and the major active site of its enzyme are shown in Figure 1.

### 2.2 Regulatory pathway of Prdx6 expression

The expression of Prdx6 is ubiquitous across all mammalian organs and tissues (Fisher, 2011). However, the expression varies across different tissues and cell types, exhibiting elevated levels in lung endothelial and epithelial cells, hepatocytes, leukocytes, and neutrophils (Patel and Chatterjee, 2019; Ambruso et al., 2012). This disparity arises from the diverse stimulation factors influencing Prdx6 across various tissues and cells. The expression of Prdx6 is modulated by various factors, including reactive oxygen species (ROS), transcription factors such as Nrf2, HIF, AP-1, NF-κB, c-Myc, C/EBP and FOXO3, as well as other enzymes and immune modulators (Sharapov et al., 2019). The expression of Prdx6 is primarily influenced by ROS. Hyperoxia and the oxidant H_2_O_2_ can induce upregulation of Prdx6 expression through augmentation of intracellular ROS levels (Kim et al., 2003). However, it has also been demonstrated that H_2_O_2_-induced oxidative stress can result in a substantial decrease in the expression level of Prdx6 (Chen et al., 2023). The antioxidant response element (ARE), a consensus sequence positioned between positions −357 and −349 in the Prdx6 promoter, plays a pivotal role in regulating both the basal transcription of Prdx6 and its response to conditions of oxidative stress (Chowdhury et al., 2009). Under conditions of oxidative stress, ROS interact with Keap1 (Kelch-like ECH-associated protein 1) and dissociate Nrf2, leading to its subsequent phosphorylation and translocation into the nucleus for transcriptional activation of Prdx6 by binding to ARE (Bellezza et al., 2018; Chowdhury et al., 2014; Chowdhury et al., 2009). However, research indicates that an excessive level of oxidative stress can result in the upregulation of Klf9 (Kruppel-like factor 9) through aberrant activation of Nrf2, leading to a significant decrease in Prdx6 mRNA expression (Chhunchha et al., 2022). And the activation of TGFβ by ROS may inhibit the NF-κB-mediated transcriptional regulation of the Prdx6 gene through a Smad3-mediated inhibitory signaling pathway, thereby suppressing the expression of Prdx6 (Fatma et al., 2009).

In addition, the direct targeting of the mouse gene Prdx6 by miR-24, which fine-tunes Prdx6 levels, has been experimentally validated. And the interaction between miR-24 and Prdx6 modulates mitochondrial ROS and DNA damage pathways to preserve satellite cell viability and myogenic potential (Soriano-Arroquia et al., 2021). It also has been demonstrated that glucocorticoids can induce the expression of Prdx6 in lung epithelial cells (Kim et al., 2002).

## 3 Biological function of Prdx6

### 3.1 Prdx6 and oxidative stress

Oxidative stress is characterized by an imbalance between the production of oxidants and the protective mechanisms provided by antioxidant defense systems, resulting in deleterious effects on biological systems (Oke et al., 2024; Forman and Zhang, 2021). And the occurrence of oxidative stress is intimately linked to an excessive accumulation of ROS (Pisoschi and Pop, 2015). Under physiological conditions, ROS serve as crucial intracellular signaling molecules that mediate diverse cellular signaling pathways involved in various biological processes (Chen et al., 2008), which participates numerous essential cellular functions, including cell growth, specialization, programmed cell death, tissue healing mechanisms, and immune response (Ray et al., 2012; Domazetovic et al., 2017). ROS represent a category of oxygen-containing compounds characterized by their highly reactive nature. It consists of both radical species, such as superoxide (O_2_^−^) and hydroxyl radicals (HO·), as well as non-radicals like H_2_O_2_ (Batty et al., 2022). Being the most stable among them, H_2_O_2_ possesses a significantly prolonged half-life and exhibits the ability to effectively traverse biofilms, thereby enabling its active participation as a signaling molecule (Dzubanova et al., 2024). The generation of ROS involves a diverse array of cellular enzyme systems, although the NADPH oxidase (NOX) family is recognized as the primary source of ROS production in the bone marrow (Dzubanova et al., 2024), the mitochondrial respiratory chain is widely acknowledged as the predominant source of ROS in most mammalian cells (Zhang and Gutterman, 2007). A minor proportion of the molecules within the mitochondrial respiratory chain, approximately 1%–4%, undergo incomplete reduction, leading to the formation of O_2_^−^ and the subsequent generation of other reactive oxygen species via various enzymatic or non-enzymatic reactions (Zhang and Gutterman, 2007). It is currently known that the occurrence and progression of various human diseases, including musculoskeletal disorders, are closely associated with oxidative stress (Song et al., 2024; Lupu et al., 2024; Xia et al., 2024; Xiong et al., 2024; Wang W. et al., 2024).

The group of enzymes involved in combating oxidative stress includes superoxide dismutase (SOD), glutathione peroxidase (GPX), glutathione reductase, glutathione S-transferase, catalase (CAT), thioredoxin reductase, heme oxygenase-1 (HO-1), ubiquinone oxidoreductase, and Prdxs (Lupu et al., 2024). Prdx6 plays a crucial function in preserving the balance of redox levels in mammalian cells by effectively diminishing various types of peroxides present within cellular surroundings (Chen et al., 2024). Upon TLR4 stimulation, the translocation of Prdx6 to mitochondria occurs. Through its TRAF-C domain, Prdx6 interacts with tumor necrosis factor (TNF) receptor-associated factor 6 (TRAF6) and forms a complex that impedes the binding of ECSIT (evolutionarily conserved signaling intermediate in Toll pathways) to TRAF6 within mitochondria, effectively suppressing mitochondrial reactive oxygen species (mROS) production (Min et al., 2017). In addition, study has demonstrated that Prdx6 overexpression can mitigate H_2_O_2_-induced damage in RPE cells by activating the EGFR/ERK pathway, thereby exerting an antioxidative stress effect and safeguarding against oxidative stress-induced cellular injury (Chen et al., 2023). Although Prdx6 has also been found to facilitate the assembly and activation of NOX2 via its PLA2 activity in astrocytes, thereby inducing the generation of ROS, this phenomenon has not been definitively demonstrated in other cell types (Peng et al., 2024; Chatterjee et al., 2011). Overall, the role of Prdx6 in scavenging excessive ROS and alleviating oxidative stress is indispensable overall.

### 3.2 Prdx6 and inflammation

As we all know, the inflammatory response represents a pathological process in which the body’s defense mechanism is activated in response to various damaging factors (Chen et al., 2018). But on the other hand, an excessive or prolonged response can give rise to a range of inflammatory disorders, including musculoskeletal conditions (Cadelano et al., 2024; Zhu et al., 2024; Zhou Y. et al., 2016; Newton and Dixit, 2012). Lots of injuries induce the release of cytokines (IL-1β, IL-6, and TNF-α) and their subsequent binding to corresponding receptors, thereby initiating crucial inflammatory signaling pathways including NF-κB, MAPK, JAK-STAT and ect, ultimately leading to detrimental inflammatory responses (Chen et al., 2018).

By suppressing the TLR4/NF-κB signaling pathway, Prdx6 demonstrates potential in mitigating the inflammatory response induced by elevated glucose levels (Wu et al., 2023). Prdx6 can effectively attenuate the levels of inflammatory mediators, including TNF-α, IL-6, and MCP-1 in cellular systems, thereby retarding the onset of inflammatory responses (Wu et al., 2023). Prdx6 exerts inhibitory effects on peripheral immune cell infiltration and neuroinflammation by downregulating the expression of Matrix metalloproteinase (MMP) 9 (which plays a pivotal role in promoting disruption of the blood-brain barrier (BBB) among various Matrix metalloproteinases (MMPs) associated with central nervous system (CNS) inflammation (Dubois et al., 1999)), as well as chemokines and free radical stress (Yun et al., 2015). Furthermore, the direct action of Prdx6 on the synthesis of regioisomers promotes the production of hydroxy fatty acids (Fatty acid esters of hydroxy fatty acids (FAHFAs) are lipid mediators exhibiting promising antidiabetic and anti-inflammatory properties, thus holding great potential for therapeutic applications), ultimately functioning as an agent with both anti-inflammatory and anti-diabetic effects (Kuda et al., 2018). Taken together, Prdx6 exhibits a pronounced anti-inflammatory function by attenuating the production of inflammatory mediators and diminishing levels of ROS, thereby potentially play a pivotal role in ameliorating inflammatory responses associated with diverse diseases, including MSDs.

### 3.3 Prdx6 and cell death

#### 3.3.1 Apoptosis and autophagy

Apoptosis and autophagy are both forms of programmed cell death that play crucial roles in maintaining normal cellular processes and tissue homeostasis (Long et al., 2024). Apoptosis, also known as type I programmed cell death and the earliest form of programmed cell death identified in humans, is characterized by DNA fragmentation, condensation of chromatin, and formation of apoptotic bodies (Erekat, 2022; Kerr et al., 1972). Autophagy is a cellular mechanism that involves cells engulfing and isolating cytoplasmic components and damaged organelles, followed by the degradation of their specific components within lysosomes (Rabinowitz and White, 2010). Mitochondrial dysfunction and abnormal extracellular matrix (ECM) metabolism can result in aberrant apoptosis and autophagy of cells, which are implicated in the pathogenesis of MSDs (Salucci et al., 2022; Ungsudechachai et al., 2024; Zheng et al., 2024; Dai et al., 2024). Besides this, inflammatory cytokines such as IL-1β promote chondrocyte apoptosis and autophagy (Lv et al., 2024).

The activation of the phosphatidylinositol 3-kinase (PI3K)/protein kinase B (AKT)/signaling pathway has been demonstrated to exert both anti-apoptotic and anti-autophagic effects (Iksen and Pongrakhananon, 2021; Liu et al., 2024). Prdx6 also can regulate the PI3K/AKT signaling cascade to mitigate H_2_O_2_-triggered cell death in ARPE-19 cells (Zha et al., 2015). The expression of Prdx6/PI3K/AKT is upregulated by circ-CARD6 through the modulation of miR-29b-3p, leading to the reduction of cellular apoptosis and autophagy (Fan et al., 2024). In addition, it has been discovered that Prdx6 suppressed TLR4/NF-κB signaling pathway to mitigate apoptosis in HK-2 cells exposed to high glucose (HG) (Wu et al., 2023).

#### 3.3.2 Ferroptosis

Unlike autophagy, apoptosis, and other types of necrosis, ferroptosis is an iron-dependent non-apoptotic cell death that is typified by the build-up of lipid ROS(87). The majority of its morphological characteristics are changes to the mitochondria, for example, there is an increase in membrane density, a notable reduction in the size of mitochondria, and either a reduction or absence of mitochondrial cristae (Li et al., 2020). As we are know, ferroptosis has been linked to numerous disorders of the kidneys, heart, brain, and tumors in addition to brain degenerative conditions like Parkinson’s, Alzheimer’s, and Huntington’s illnesses (Stockwell et al., 2017). Additionally, it is well-established that ferroptosis plays a significant role in MSDs such as OP, OA, RA, and IVDD (Zhang Y. et al., 2022). Lipid metabolism, iron metabolism, and system X_c_^−^/glutathione peroxidase 4 (GPX4) are the three main processes implicated in ferroptosis (Zhang M. et al., 2024). Aberrant intracellular iron metabolism results in excessive iron levels, which contribute to lipid peroxidation via the Fenton reaction, and at the same time, the loss of GPX4 activity by system X_c_^−^ inhibition or other reasons eventually leads to the accumulation of lipid peroxides, which damages cells and leads to ferroptosis (Feng and Stockwell, 2018; Li and Li, 2020). As a regulated form of cell death, ferroptosis can be inducted by inducing agents, such as erastin, RSL3, other DPI compounds, sorafenib, FIN56 (Dixon et al., 2012; Yang et al., 2014; Dixon et al., 2014; Shimada et al., 2016). The mechanisms of are broadly categorized as inhibition of system X_c_^−^, removal of glutathione, and direct inhibition of GPX4. Ferroptosis is known to be regulated by a range of pathways such as the glutaminolysis, transsulfuration, and mevalonate pathways (Yang and Stockwell, 2016).

**FIGURE 2:**
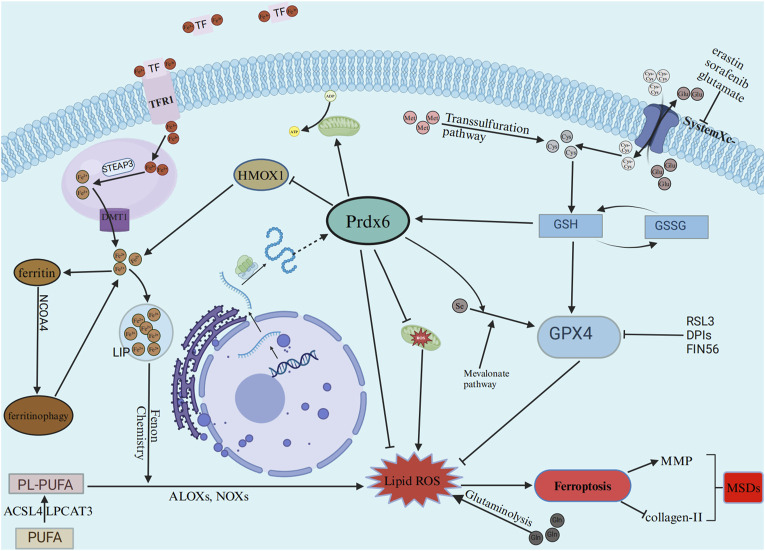
The effects of ferroptosis on musculoskeletal diseases are mitigated by Prdx6 through the reduction of free iron levels, lipid peroxide levels, and inhibition of ROS production caused by mitochondrial damage. TF, transferrin; TFR1,transferrin receptor; STEAP3, STEAP3 metalloreductase; DMT1, divalent metal transporter 1; LIP, labile iron pool; HMOX1, heme oxygenase 1; GPX4, glutathione peroxidase 4; PUFA, polyunsaturated fatty acid; PL-PUFA, phospholipids rich in polyunsaturated fatty acids; ACSL4, acyl-coA synthetase long chain family member 4; LPCAT3, lysophosphatidylcholine acyltransferase 3; ALOXs: Arachidonate-lipoxygenases; NOXs, nicotinamide adenine dinucleotide oxidases; GSH, glutathione; GSSG, glutathione, oxydized.

Research has demonstrated that Prdx6’s phospholipase, LPCAT, and peroxidase activities are all helpful to the restoration of membrane lipid peroxidation (Fisher et al., 2018). By conducting functional analyses of the AIPLA2 inhibitor MJ33 and the GPX4 inhibitor ML210, as well as analyses of their co-dependency, researchers revealed that GPX4 and Prdx6 exhibit a positive functional correlation and act synergistically in the repair process of oxidized phospholipids (Paluchova et al., 2022; Santesmasses and Gladyshev, 2022). Subsequent study in cells derived from animals with Prdx6 and GPX4 knockdown revealed that cells could compensate for the loss of Prdx6 through the upregulation of GPX4, and conversely, the deficiency of GPX4 could be alleviated by enhanced expression of Prdx6. Nevertheless, Prdx6-deficient pulmonary endothelial cells remained vulnerable to ferroptosis (Torres-Velarde et al., 2024). Another study showed that overexpression of Prdx6 increased the expression of SLC7A11 and GPX4 in Mouse glomerular podocytes MPC5, which ultimately inhibited HG-induced ferroptosis (Zhang et al., 2021). These results further highlight a distinct interplay between GPX4 and Prdx6 in the regulation of cell ferroptosis. On the other hand, Prdx6 has the ability to enhance mitochondrial function (Torres-Velarde et al., 2024). The function of HMOX1 was also inhibited by Prdx6, resulting in a reduction in iron content and a decrease in the occurrence of the Fenton chemical reaction (Lu et al., 2019). Furthermore, *in vitro* and *in vivo* experiments showed that Prdx6 is a selenium carrier protein, and its cysteine residue 47 plays a key role in selenium utilization, which promotes selenium phosphate synthetase 2 (SEPHS2) to maintain GPX4 expression levels more efficiently, thereby regulating cell sensitivity to ferroptosis (Fujita et al., 2024; Ito et al., 2024). Interestingly, Prdx6 was found to be expressed in the musculoskeletal system and decreased in MSDs (Ikeda et al., 2013; Guillén et al., 2021; Kawaai et al., 2024). Based on the aforementioned findings, we hypothesized that Prdx6 could inhibit ferroptosis through multiple mechanisms: directly reducing lipid peroxidation products and indirectly modulating lipid peroxides via its influence on lipid metabolism, iron metabolism, mitochondrial function, and interaction with GPX4. This process may ultimately be effective in delaying MSDs. Taken together, Prdx6 may play an important role in the occurrence and development of ferroptosis. Prdx6 can slow down cell ferroptosis through a variety of mechanisms, including reducing intracellular free iron ions to inhibit Fenton chemical reaction, maintaining mitochondrial function to reduce mitochondrial damage, directly reducing the level of lipid peroxidation products, and increasing the level of selenoprotein, so as to delay the progression of metabolic diseases. The associated pathways implicated in the inhibition of ferroptosis by Prdx6 are depicted in Figure 2.

## 4 The potential roles of Prdx6 in musculoskeletal diseases

### 4.1 Osteoporosis

Osteoporosis is a degenerative bone disease of the musculoskeletal system characterized by a reduction in bone mineral density and the deterioration of bone microstructure, resulting in heightened bone fragility and an increased susceptibility to fractures (Sun et al., 2022). Primary osteoporosis and secondary osteoporosis are the two main categories of osteoporosis (Wu et al., 2024; Miyakoshi et al., 2017). In recent years, there has been a significant increase in the prevalence of OP due to population aging (Feng et al., 2018; Elahmer et al., 2024). The exact cause of OP remains unclear, but it is believed to be influenced by various factors such as aging, genetics, inadequate consumption of vitamin D and calcium, sedentary lifestyle, menopause in women, excessive alcohol intake and inflammation (Kim and Kim, 2019).

Bone is a dynamic tissue that undergoes constant remodeling, with osteoclasts being involved in the breakdown of old bone while osteoblasts contribute to the formation of new bone (Schröder, 2019). The process of bone remodeling involves a diverse range of local factors (such as TGF-β, macrophage colony-stimulating factor (M-CSF), NF-κB ligand receptor activator (RANKL)), systemic regulatory factors (including vitamin D, PTH, estrogen, glucocorticoids and ect), and metalloproteinases (Li et al., 2021b). The disruption of this equilibrium, with an excessive increase in bone resorption compared to formation, represents one of the primary etiological factors contributing to OP (Sun et al., 2022). This review highlights that Prdx6 possesses the ability to attenuate local inflammatory factors, M-CSF and MMPs, while also inhibiting the activation of the NF-κB pathway. Consequently, Prdx6 may exert a pivotal role in mitigating bone degradation and maintaining optimal bone metabolism balance. Research has shown that excessive iron levels can augment osteoclast-mediated bone resorption via the RANK/RANKL pathway and exert detrimental effects on osteoblasts and bone formation by repressing the osteogenic differentiation potential of MC3T3 cells and bone marrow mesenchymal stem cells (BMSCs), ultimately contributing to the pathogenesis of OP (Yang Y. et al., 2023; Gao et al., 2022). Prdx6 can attenuate excess iron production by inhibiting HMOX1, and effectively reduce the levels of lipid peroxidation products thereby inhibiting ferroptosis. These effects may mitigate osteoblast damage in OP. The role of inflammation in osteoporosis is also significant. Inflammatory cytokines primarily impact bone metabolism through the RANK/RANKL/OPG pathway, thereby promoting the development of osteoporosis (Barbour et al., 2012). ROS triggers the activation of receptor activator of NF-κB ligand (RANKL) and TNFα through ERK and NF-κB pathways, thereby promoting osteoclastogenesis and bone resorption both *in vitro* and *in vivo*. Inversely, bone marrow-derived monocytes/macrophages differentiate into osteoclasts, which further enhance ROS production via a cascade involving TNFα, TRAF6, Rac1, and nicotinamide adenine dinucleotide phosphate oxidase (NADPH) (Manolagas, 2010). Furthermore, previous studies have demonstrated that metabolic abnormalities in diabetes result in heightened levels of ROS within cells (Giacco and Brownlee, 2010), which largely contribute to the development of diabetes-associated osteoporosis. Prdx6 utilizes its associated enzymatic activity to effectively eliminate excessive ROS within the body, it also can enhance mitochondrial function and mitigating mitochondrial damage to reduce ROS production (Torres-Velarde et al., 2024). Consequently, Prdx6 may play a crucial role in maintaining bone homeostasis and ultimately preventing the onset of osteoarthritis by diminishing ROS levels in bone tissue, minimizing macromolecular damage, and alleviating dysfunction and injury in OP. Additionally, Prdx6 modulates the REDOX status and thereby enhances osteogenesis while promoting fresh bone formation (Kawaai et al., 2024), and it exhibits the potential to reduce disulfide bonds in extracellular matrix proteins within calcified cartilage, ultimately facilitating osteoblast development through its osteogenic activity (Kawaai et al., 2024).

Overall, based on the aforementioned effects of Prdx6 on oxidative stress, inflammation, and cell death, it is postulated that Prdx6 may effectively mitigate ROS, ameliorate the deleterious impact of inflammatory mediators, impede bone mass loss induced by ferroptosis, apoptosis, and autophagy in chondrocytes, and attenuate bone resorption. Therefore, Prdx6 may contribute to the maintenance of bone homeostasis through the aforementioned mechanisms, thereby ultimately ameliorating the deleterious effects of OP on bone and joint integrity.

### 4.2 Osteoarthritis

OA is a prevalent musculoskeletal condition characterized by the degeneration of articular cartilage, thickening of subchondral bone, development of osteophytes, and varying levels of synovial inflammation (Chen et al., 2017). Among them, cartilage degeneration is considered the foremost pathological alteration, and the morphology, structure, and alterations in articular cartilage composition serve as crucial diagnostic basis (Zhang H. et al., 2024). The typical clinical manifestations observed in patients with OA encompass pain, stiffness, joint swelling, and limitations in joint function (Shentu et al., 2024). The etiology of OA is multifaceted, encompassing various risk factors such as advanced age, genetic predisposition, joint trauma, excessive body weight, chronic inflammation, joint misalignment, and structural abnormalities (Chen et al., 2017; Loeser et al., 2012). With the global population experiencing growth and aging, there has been a substantial increase in the prevalence of OA (GBD, 2021 Osteoarthritis Collaborators, 2023). Currently, the management of OA primarily relies on conservative treatment and joint replacement surgery; however, these therapeutic approaches do not offer complete prevention against cartilage degeneration or halt OA progression, while also exhibiting evident adverse effects (Shentu et al., 2024; Li et al., 2023). Therefore, the search for a novel and efficacious therapeutic target for OA is of utmost urgency.

It is widely acknowledged that oxidative stress and inflammation play a significant role in the pathogenesis and progression of OA (Loeser et al., 2016; Kang et al., 2022). ROS and its oxidation products can induce chondrocyte apoptosis by regulating the PI3K/AKT, JNK, and NF-κB signaling pathways, upregulate the expression of metalloproteinases (MMP-1, MMP-3, and MMP-13, etc.) in the synovium, control the inflammatory response, and disrupt the mitochondrial pathway, leading to synovial damage (Lepetsos and Papavassiliou, 2016; Vaillancourt et al., 2008). In addition, it downregulates the expression of metabolic genes related to cartilage synthesis, such as aggrecan (ACAN) and collagen type II α1 (COL2A1), ultimately impairaging chondrocyte function and joint integrity (Liu et al., 2023). As mentioned earlier, Prdx6 may lead to a reduction in the levels of ROS and its oxidation products, such as lipid peroxidation products, which may mitigate the occurrence of this deleterious process. In addition, Prdx6 may mitigate the deleterious effects of ROS on cartilage and synovium by activating the PI3K/AKT survival pathway, inhibiting the activation of the NF-κB signaling pathway, and inhibiting MMPs. Previous study has shown that the regulatory factor Toll-like receptor 4 (TLR4) governs the NF-κB signaling pathway, facilitating the sequestration of NF-κB from IκB and its translocation into the nucleus for the regulation of inflammatory cytokine expression, initiation of joint destruction, and progression of OA (Ding et al., 2019). Various factors, including mechanical stress, trauma, aging, and metabolic disorders, can induce chondrocytes to release inflammatory cytokines (IL-1β、TNF-α、IFNγ and ect). Subsequent activation of NF-κB by these cytokines promotes catabolism and leads to cartilage degradation. This further exacerbates mechanical stress and joint damage while perpetuating inflammation within the synovial space, thereby establishing a detrimental positive feedback loop involving sustained NF-κB activity (Arra et al., 2020). The aberrant activation of NF-κB binds to the binding site situated within the promoter region of interleukin-1β (IL-1β) gene, thereby eliciting an inflammatory response and facilitating OA progression (Li et al., 2014). The Prdx6 not only mitigates chronic inflammation induced by ROS and MMPs, but also suppresses NF-κB activity through downregulating inflammatory factors like IL-1β and TNF-α, thereby attenuating the inflammatory response. IL-1β can promote the production of matrix MMPs by chondrocytes, inhibit the expression of collagen type II with cartilage characteristics, promote the production of collagen type I with fibroblast characteristics, promote the production of NO, inhibit the synthesis of collagen and proteoglycan, promote cell apoptosis and other ways to damage cartilage and facilitate the onset of OA (Shentu et al., 2024). Prdx6 exhibits a pronounced potential for mitigating cartilage injury by reducing IL-1β, MMPs, and inhibiting apoptosis. Furthermore, despite the incomplete elucidation of the underlying mechanism of ferroptosis in OA, numerous researchers have reported its innegligible role in driving OA progression (Yao et al., 2021; Zhang S. et al., 2022). The iron concentration in synovial fluid has been observed to be elevated in patients with OA, and the induction of chondrocyte ferroptosis also leads to an upregulation of matrix MMP13 and a downregulation of collagen II expression (Yao et al., 2021; Ji et al., 2024), but Prdx6 can reduce excess iron as previously described. Moreover, it has been reported that Prdx6 suppressed the production of pro-inflammatory cytokines and conferred protection to OA chondrocytes against IL-1β-induced oxidative stress by mediating adipose-derived mesenchymal stem cell (AD-MSC)-derived microvesicles (MV) and enhancing the expression of autophagy marker LC3B (Guillén et al., 2021).

According to the above, Prdx6 exhibits significant antioxidative stress, anti-inflammatory response, and anti-ferroptotic functions. Therefore, we postulated that Prdx6 may exert a pivotal beneficial role in OA by attenuating ROS generated through oxidative stress, ameliorating the inflammatory response within bone and joint tissues, and suppressing chondrocyte ferroptosis. This presents an opportunity to discover novel therapeutic targets for OA.

### 4.3 Intervertebral disc degeneration

The IVD, as the largest avascular tissue in the human body, is comprised of the central nucleus pulposus (NP), the peripheral annulus fibrosus (AF), and the cartilage endplate (CEP) at its upper and lower ends, which serves to mitigate spinal pressure resulting from body weight and muscle contraction while enhancing spinal flexibility (Muñoz-Moya et al., 2024; Wang P. et al., 2024). IVDD arises from an inequilibrium in the intervertebral disc’s anabolic and catabolic metabolism, contributing significantly to the persistence of chronic LBP (Xu X. et al., 2024). This condition significantly impacts patients' quality of life and imposes a substantial personal and socio-economic burden (Mohd Isa et al., 2022; Cieza et al., 2021). So far, conservative and surgical interventions represent the prevailing approaches for managing IVDD; however, neither of these strategies addresses the fundamental issue underlying this condition (Wang et al., 2023).

Factors associated with IVDD, including environmental, genetic, and nutritional influences, contribute to alterations in cell morphology, inflammation, excessive production of ROS, increased senescence of cells, and cellular death within the IVD (Xu B. et al., 2024; Roh et al., 2021). Oxidative stress and inflammatory responses are widely acknowledged as non-negligible factors contributing to IVDD (Xu B. et al., 2024; Yu et al., 2021). The excessive production of ROS caused by mitochondrial damage is related to the pathogenesis of IVDD: it can cause nucleus pulposus cell damage or death through multiple pathways, and destroy the balance of ECM metabolism and damage the integrity of intervertebral disc by inhibiting matrix synthesis and upregulating the expression of matrix degradation protease (Feng et al., 2017). Therefore, we hypothesized that Prdx6 enzyme activity could effectively reduce oxidative stress injury in IVD, and Prdx6 could alleviate the degradation of ECM in IVD by inhibiting the enzymatic activity of MMPs. In IVDD, various cell types including NP and AF cells, as well as macrophages, T cells, and neutrophils, have the ability to secrete proinflammatory mediators such as TNF-α, IL-1α/β, IL-6, IL-8, IL-2, IL-10, IFN-γ, and chemokines (Risbud and Shapiro, 2014). Among these mediators, IL-1β and TNF-α are considered pivotal inflammatory cytokines in IVDD and have been extensively investigated (Risbud and Shapiro, 2014; Wang et al., 2020). The involvement of IL-1β and TNF-α in the initiation and progression of IVDD is well-established, as they amplify inflammatory responses, promote IVD cells senescence, accelerate apoptosis and autophagy, stimulate ECM degradation, and exacerbate oxidative stress (Wang et al., 2020). For instance, upon exposure to TNF-α and IL-1β, the NF-κB signaling pathway is stimulated, leading to an increase in the expression of MMPs, pro-inflammatory cytokines (ADAMTS-5), and FasL. These molecules play critical roles in the development and progression of IVDD. And activation of the NF-κB signaling pathway results in an upregulation of miR-640 expression, exacerbating inflammatory responses and apoptosis within nucleus pulposus cells, thereby establishing a detrimental feedback loop (Dong et al., 2019). In addition, senescent NP cells undergo senescence associated secretory phenotype (SASP) and secrete proinflammatory molecules, which can lead to local inflammation in IVD and further aggravate the degenerative process (Wang W. et al., 2024). Noteworthily, the role of Prdx6 in reducing the inflammatory response in IVD cells is potentially significant, as it effectively decreases IL-1β and TNF-α levels, and inhibits NF-κB activation. Ferroptosis has been demonstrated to occur in the NP, AF, and CEP of the IVD, leading to upregulation of extracellular matrix degradation-related enzymes, which is closely associated with the pathophysiological progression of IVDD (Zhou et al., 2022). Prdx6 may exert a protective effect on NPC, AF, and CEP by attenuating intracellular levels of free iron and lipid peroxides, as well as interacting with GPX4 to mitigate ferroptosis. In addition, activation of the Keap1-Nrf2-ARE signaling pathway can potentiate the expression of diverse antioxidant gene clusters, attenuate oxidative stress within the IVD, safeguard nucleus pulposus cells, and ultimately effectively retard IVDD (Song et al., 2021). Interestingly, activation of the Keap1-Nrf2-ARE signaling pathway can induce an upregulation in Prdx6 gene expression. The implication here is that the Prdx6 gene may constitute one of the antioxidant genes involved in this particular process.

Therefore, Prdx6 may mitigate the progression of IVDD by modulating the antioxidative stress response, exerting an anti-inflammatory effect on the IVD, and attenuating IVD cell apoptosis, thereby demonstrating potential therapeutic advantages for IVDD.

## 5 Inducers of Prdx6 and their potential effects

### 5.1 Curcumin

Curcumin (CUR), the bioactive compound derived from Curcuma longa, exhibits potent anti-cancer, anti-apoptotic, anti-inflammatory, and antimicrobial properties (Chhunchha et al., 2013). Given its extensive range of pharmacological effects and ability to target multiple molecules, this compound has substantial implications for the prevention and treatment of various diseases, including musculoskeletal disorders (Yang S. et al., 2023; Zhao et al., 2024; Ding et al., 2024; Yaikwawong et al., 2024; Zhang C. et al., 2024). Currently, CUR has garnered relatively more attention as an inducer of Prdx6 in scientific research. CUR can enhance the expression of Prdx6 through SP1-mediated upregulation, thereby mitigating aberrant oxidation products and exhibiting significant antioxidant function (Jia et al., 2017), and mitigating endoplasmic reticulum stress induced by ROS and aberrant NF-κB signaling, ultimately safeguarding cells against hypoxia-induced oxidative stress and ER stress (Chhunchha et al., 2013; Chhunchha et al., 2011). Moreover, CUR exhibits the ability to attenuate NF-κB signaling pathway activation by upregulating Prdx6 and inhibiting P65 nuclear translocation, which is beneficial to reduce the damage caused by inflammatory response (Li et al., 2022). In addition, it has been reported that CUR can inhibit autophagy-dependent ferroptosis through Sirt1/AKT/FoxO3a signaling in cardiomyocytes (Zhao ST. et al., 2025).

The compound CUR has been discovered to possess numerous advantageous effects in the realm of MSDs. The combination of CUR and probucol exhibits inhibitory effects on chondrocyte apoptosis, reduces cartilage destruction in OA, maintains mitochondrial function and cartilage ECM stability by suppressing the PI3K/AKT/mTOR signaling pathway. And the combination of TPL and CUR can mitigate the progression of rheumatoid arthritis by suppressing the IL-17/NF-κB signaling pathway (Zhang C. et al., 2024). It promotes COL-II expression in ECM while inhibiting MMP-3 and MMP-13 expression (Han et al., 2021). Additionally, curcumin exerts a suppressive effect on bone resorption by downregulating the NF-κB/JNK signaling pathway and inhibiting osteoclast activity, thereby potentially ameliorating OA (Ding et al., 2024). Studies also show that CUR may ameliorate osteoporosis by mitigating apoptosis, oxidative stress, and inflammation, while promoting osteogenic differentiation and inhibiting osteoclastogenesis (Yang S. et al., 2023). And we assume that these beneficial effects of CUR on MSDs may be attributed to its ability to enhance the expression of Prdx6. However, although current studies have found that it has a positive therapeutic effect on MSDs, its shortcomings, such as difficulty in extraction, low solubility, rapid metabolism, and low stability, undoubtedly greatly limit its clinical application (de Souza Ferreira and Bruschi, 2019). Another study demonstrated that CUR can induce ferroptosis by promoting the ubiquitination and degradation of GPX4, a process that is inhibited by the overexpression of GPX4 (Zhao W. et al., 2025). The main reason for the contradictory therapeutic effects and limitations of CUR is that the research on its mechanism of action is not deep enough and the understanding is still relatively vague.

### 5.2 Others

With the close attention to Prdx6, many other agents and compounds have been found to upregulate the expression of Prdx6 and exert beneficial effects on body in recent years. The mechanism by which they promote Prdx6 may involve multiple pathways. The expression of the Prdx6 gene can be enhanced by date palm pollen (DPP), leading to an improvement in semen quality (Falahati et al., 2023). The expression of Sp1 and Prdx6 can be upregulated by Ginkgolic acid (GA), which in turn reduces cell damage through the attenuation of abnormal sumoylation signal transduction induced by oxidative stress and enhancement of response element enrichment within the Prdx6 promoter (Chhunchha et al., 2018). Epigallocatechin-3-gallate (EGCG) can upregulate the expression of Prdx6 via activation of the MAPK signaling cascade, thereby conferring protection against oxidative damage to vital organs (Dickinson et al., 2014). The circ-CARD6 exerts its influence on miR-29b-3p, leading to the upregulation of Prdx6 expression. Consequently, this activation triggers the PI3K/AKT pathway while concurrently reducing levels of ROS, apoptosis, and autophagy (Fan et al., 2024). Andrographolide (Andro) has been found to upregulate the expression of not only Prdx6, but also Prdx1 and Prdx4, thereby attenuating oxidative stress and ameliorating liver injury induced by LPS (Ge et al., 2023). An suitable dose of sulforaphane (SFN) effectively activates the Nrf2/ARE pathway by facilitating nuclear translocation of Nrf2, thereby upregulating Prdx6 expression to counteract age-related diseases (Kubo et al., 2017). In addition to these effects, based on the aforementioned summary in this review, it can be postulated that these Prdx6 inducers may possess potential therapeutic efficacy in MSDs through upregulating the expression of Prdx6 to modulate oxidative stress, inflammation, and cellular apoptosis pathways. The sites and associated effects of Prdx6, regulated by activators of Prdx6, are presented in Table 2.

**TABLE 2:** The inducers of Prdx6 and potential mechanisms.

Activators	Signaling pathway	Related effects
CUR	Sp1 and NF-κB	Anti-oxidative stress, anti-apoptosis and reduction of inflammation
DPP	Nrf2	Improving Semen quality
GA	Sp1	Anti-oxidative stress
EGCG	MAPK	Anti-oxidative stress
circ-CARD6	PI3K/Akt	Decreased ROS, apoptosis and autophagy
SFN	Nrf2/ARE	Fighting age-related diseases
Andro	MAPK	Alleviating oxidative stress and improving LPS-induced liver injury

CUR, curcumin; DPP, date palm pollen; GA, ginkgolic acid; EGCG, epigallocatechin-3-gallate; SFN, sulforaphane; Andro, andrographolide.

## 6 Challenges and future directions

Although the current study indicates that Prdx6 may serve as a potential target for effective therapeutic relief of MSDs, there are still many challenges to truly realize this benefit. For example, studies have shown that long-term overexpression of Prdx6 may promote tumor growth or may have carcinogenic effects, and the small number of blood vessels in the musculoskeletal system and the difficulty of drug penetration make it difficult to regulate the maintenance of Prdx6 expression. In addition, many studies on Prdx6 activators (such as CUR) are limited to animal models, lacking sufficient clinical trials to verify their findings, and their activators still have many disadvantages (such as difficulty in extraction, low solubility, fast metabolism, low stability, and bioavailability) in clinical application. Based on the above challenges, more attention should be paid to the precise regulation of Prdx6 in future research. Specifically, more advanced bone, cartilage and IVD-specific delivery technologies can be developed to clarify the specific mechanism of Prdx6 in different stages of MSDs and other diseases, so as to effectively reduce their systemic reactions. In addition, based on the existing research basis, the efficiency of CUR in MSDs application can be further improved through nanotechnology related strategies.

## 7 Summary and prospects

**FIGURE 3:**
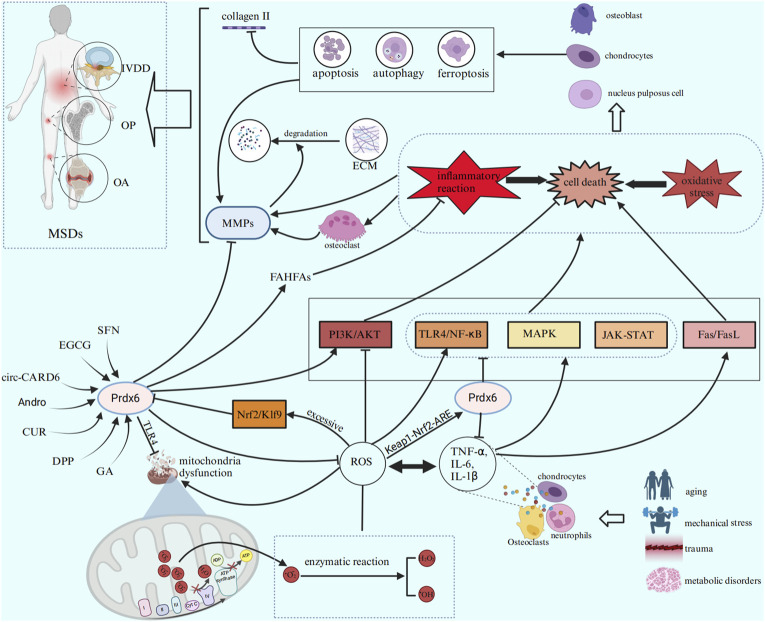
Prdx6 inducers can enhance the expression level of Prdx6 and exert a potential role in ameliorating MSDs by inhibiting inflammation, cell death, and oxidative stress through various signaling pathways.

The significant roles of Prdx6 have gained increasing recognition in recent years, encompassing its antioxidative stress and anti-inflammatory properties, as well as its capacity to mitigate aberrant cell death. Numerous studies have demonstrated its significant therapeutic potential in various systems, encompassing tumors, vascular diseases, chronic inflammatory disorders, and age-related ailments. This review provides an overview of the structure, expression regulation, anti-inflammatory and anti-oxidative properties, as well as the mechanisms underlying cell death prevention by Prdx6. Additionally, we discuss the positive role of Prdx6 in the pathogenesis of OP, OA, and IVDD, enumerating certain inducers of Prdx6 and their potential contribution to MSDs through upregulation of Prdx6. In conclusion, Prdx6 may reduce the damage, apoptosis and ferroptosis of osteoblasts, chondrocytes and nucleus pulposus cells by alleviating oxidative stress and inflammation. Thus, it can reduce bone degradation and maintain the optimal bone metabolism balance in OP, delay the occurrence of articular cartilage degeneration and synovitis in OA, and reduce the IVDD and the degradation of extracellular matrix in IVD. The main regulatory effects of Prdx6 in MSDs are summarized in Table 3. Finally, we speculate that Prdx6 is expected to become a new therapeutic target for MSDs by reducing the common important pathogenic factors of MSDs (oxidative stress, inflammation, ferroptosis, apoptosis and autophagy). The potential role of Prdx6 in the pathogenesis of MSDs is illustrated in Figure 3. Therefore, Prdx6 may offer a promising avenue for alleviating the pain and mitigating the social and economic burden experienced by patients afflicted with MSDs. However, there is still a lack of research on the expression of Prdx6 in the musculoskeletal system and its specific positive regulatory role in MSDs through oxidative stress, inflammation and cell death. In addition, the optimal activator of Prdx6 and how to achieve therapeutic effects by upregulating its expression have not been clarified. Based on previous findings, we propose the following hypotheses: Prdx6 has expression activity in the musculoskeletal system, and its activator may effectively regulate the expression of Prdx6 at the gene level and upregulate the expression level of Prdx6. It further regulates the key molecules and cellular events involved in the occurrence and development of MSDs, such as ROS, inflammatory factors, MMPs, collagen metabolism, ferroptosis, cell senescence, apoptosis, and autophagy, so as to delay the progress of MSDs at the molecular and cellular levels. Here, we hope that this review will provide new ideas and references for relevant researchers, and also hope that more researchers will study the specific mechanism of Prdx6 in musculoskeletal diseases, find its ideal target drugs, and write a new chapter for the treatment of MSDs.

**TABLE 3:** The main regulatory effects of Prdx6 in MSDs.

MSDs	The main role of Prdx6	Related effects	Results
OP	Inhibition TGF-β, ROS, M-CSF, MMPs, NF-κB pathways, iron, peroxidation products levels, HMOX, and regulation of REDOX status	Improve oxidative stress, inflammatory response, reduce osteoblast damage, apoptosis and ferroptosis	Reduce bone degradation and maintain optimal bone metabolic balance
OA	Inhibition HNE, IL-1β, TNF-α, iron, ROS levels, NF-κB pathways; activation of the PI3K/AKT survival pathway	Anti-oxidative stress, reduction of inflammation, reduce chondrocytes damage, apoptosis and ferroptosis	Delay the degeneration of articular cartilage and synovitis
IVDD	Decreased levels of IL-1β, TNF-α, iron, ROS, MMPs and peroxidation products levels; inhibition of NF-κB	Reduce inflammatory response, oxidative stress cellular senescence, apoptosis, and ferroptosis in nucleus pulposus cells	Alleviate IVD degeneration and extracellular matrix degradation
